# Shock

**Published:** 1888-11

**Authors:** David W. Cheever

**Affiliations:** Boston


### SHOCK.*

BY DAVID W. CHEEVER, M. D., OF BOSTON.

The operative surgery of our time has annulled pain temporarily, arrested hemorrhage permanently, averted septic absorption. It has not prevented shock. This is still a cause of much fatality. It is the object of this paper to inquire whether modern surgical procedure has *diminished* shock, wherein it fails to do so, and to suggest improvements of its defects.

What is shock?

When any one gropes his way in a dimly lighted passage and meets unexpectedly a strange person at some turning, he experiences a start, a mental apprehension, his heart turns over, flutters, but at once recovers its balance. Pursuing his path, if he now, in descending, misses a step in the dark, he has a greater shock to his nerves, he braces himself, flutters, sweats, or is chilled. If he falls and bruises himself moderately, he has vertigo, nausea, cold sweat, pain. If he falls and breaks open a joint, he has syncope, epileptiform convulsions, nausea, fluttering pulse, sweat, pain. If he injures himself more severely, he has unconsciousness.

---

* Read before the American Surgical Association at the First Triennial Congress of American Physicians and Surgeons, at Washington, September 20, 1888.—*Boston Medical and Surgical Journal.*

This is a simple description of the degrees of shock:

Apprehension,
Fluttering,
Sweating,
Chilliness,
Pain,
Vertigo,
Nausea,
Faintness,
Convulsions,
Unconsciousness,
Collapse.

The phenomena of a fainting fit are the phenomena of shock. Sudden, disagreeable, painful, destructive impressions produced on the surface or efferent nerves, and affecting the brain; thence the ganglionic system; then the heart, the stomach, the skin; and thus the brain, at last.

Moderate shock terminates in reaction. This is the *recoil* of the system. It restores the balance; but the pendulum which marks the nervous force swings back beyond the normal line.

We have temporary fever, flush, full pulse, excitement.

Severe shock is more lasting. The pulse vibrates, intermits, flags, rallies, flags again, is soft, compressible, uncertain; faintness is constant, but partial; vomiting occurs; cold extremities; dilatable pupils; pallor; imperfect reaction; very slow recovery; a condition where a feather turns the scale against the patient.

If now an operation is done, we have renewed shock, prolonged shock, secondary shock; a matter of days rather than hours; persistent nausea; exhaustion; lowered temperature; diarrhœa; imperceptible and gentle death. Or, if in an old person, that state known as prostration with excitement; typhoidal delirium, a dusky flush over the malar bones, dull eyes, intermittent pulse, jactitation, exhaustion, death.

Primary shock: reaction; early and perfect; or slow and imperfect. Secondary shock: prostration, nausea, excitement, collapse. Loss of blood, from accident or operation, adds to the shock or complicates its symptoms.

Jar, crushing, mutilation, pain, cutting, bleeding, chilling, all act on the nervous center; react on the ganglia, the heart, the power of breathing, the temperature, the consciousness, the life.

Given then the problem and the phenomena of shock, what particular influences have the operative procedures of *modern* surgery upon them?

They may be summed up in three points:

The effects of anæsthetics;
The effects of the operations;
The effects of the dressings.

These all belong together and affect each other.

Anæsthetics annul pain, but end in nausea.

Operations under anæsthetics are needlessly prolonged and exhausting.

Modern dressings are tedious and chilling.

Have we lessened or added to shock by modern surgery?

Pain and bleeding are *less*. Slow cutting, nausea, exposure, low temperature, are *more*. Primary shock is diminished; secondary shock is increased.

Formerly the time consumed in an operation was short. An amputation was hurried, now it is deliberate; an abscess was incised, now it is aspirated and curetted; a joint injury was cut off, now it is excised; the peritoneum was peeped into, now it is boldly explored; the bladder was cut for stone, now it is a prolonged crushing and washing; a breast was amputated, now the axilla is formally dissected. The old method was a matter of minutes; now it is one of hours.

Patients are frequently from one and a half to two hours on the operating table, and three hours in recovering consciousness so that they can swallow. Do we realize what this prolonged cutting, pinching and dissecting mean to the nervous system after anæsthesia is past? Does not the long exposure of the great veins to the air, in dissecting tumors, increase coagulability and future infarction? Can the peripheral nerves be lacerated *seriatim* without exhausting their constitutional irritability? It is recognized that long continued and large dissections on the front and sides of the neck are especially fatal.

Operations of *secondary* magnitude are now so prolonged that I have repeatedly seen patients die of primary shock, or *repeated shocks*, where the patient was one to two hours under the knife. It is said that he had not the vitality to resist. He had not; but consider what a perineal section, scooping out a uterine tumor, curetting a bladder, removing glandular enlargements, sometimes involve in time, in exhaustion, in capillary oozing, in shock.

Equally unphilosophical and fatal is the practice of operating in cases of primary shock before reaction has come on. An amputation is begun in half-life and ended in death. Especially difficult to decide are the cases where the patient reacts imperfectly and relapses. These cases are easily made fatal, and only saved by quick amputations, slight exposure and short anæsthesia. The golden moment of fairly established reaction must be seized before traumatic fever sets in. This moment comes in from six to eighteen hours after the injury, or it never comes.

It should be considered an *axiom* that anæsthesia does not diminish existing shock, but only annuls the additional shock which the pain of cutting produces. It prevents the pain of an operation from increasing the shock which may be present from an injury. It prevents the pathological case from experiencing the shock produced by the pain of cutting out a tumor; it does *not* prevent the secondary shock of the mutilation; it adds to secondary shock if the anæsthesia is prolonged.

In feeble subjects the lack of nourishment which precedes an operation, desirable on account of safe anæsthesia, is much aggravated by their inability to retain food after the operation. This has an important influence in bringing about collapse.

Lowering of the bodily temperature is constant after an operation under anæsthesia. The thermometer frequently falls to 97°, to 96°, and after severe and prolonged operations, to 95° Fah. This is a very serious matter, and has a marked influence in delaying reaction from shock. This chilling of the vital heat is induced, first, by anæsthesia, which, if prolonged, ends in a dripping sweat; next, by careless exposure during an operation. Then also it is largely due to antiseptic irrigations, to vapor douches of similar agents, to applications of cloth wet in corrosive or carbolic solutions around the site of the operation. The axillæ, the neck, the thorax and the abdomen are especially prone to deleterious chilling in this way.

Evaporation is a great factor in reducing heat; and this is constantly occurring on the body of the patient, in a warm atmosphere, during a prolonged operation. Especially is this dangerous when the peritoneal surfaces are exposed; evaporation then is very rapid and very extensive.

Warm douches and washes give as great a subsequent chilling as cold ones, as all experience who take a tepid bath.

The sufferer is frequently allowed to lie about too long, under an anæsthetic, waiting for his turn, in busy hospital practice.

The surgical toilette of wounds, in the modern modes of dressing, is depressing, exhausting, devitalizing.

Finally comes the greatest evil of all, nausea. Nausea is one of the marked symptoms of severe shock, primary or secondary. Unfortunately, anæsthetics very frequently produce this as a secondary effect.

Persistent vomiting and retching mark the slow sinking and collapse of secondary shock after capital operations. Continued nausea is one of the worst of symptoms; begun in pain and shock, it recurs after anæsthesia, and continues as the most dangerous factor in preventing reaction.

What can we do to prevent or diminish shock?

(1) Wait for reaction.

(2) Never neglect to calm those suffering mental shock by a cheerful word and personal appearance.

(3) Give alcohol, either spirits or wine, a quarter of an hour before the anæsthetic.

(4) Make the anæsthesia short; never begin it until everything is ready; suspend it during the less painful dressings. Consciousness returns *tardily*. We keep up the anæsthetic longer than is necessary.

(5) As rapid an operation as can prudently be done.

(6) As short a dressing as is practicable.

(7) As a cardinal point, avoid *chilling* the patient.

To promote reaction after the operation:

(1) Persistent and *carefully* applied *dry* heat. (Be *over*-careful about accidental burns.)

(2) Liquid nourishment, combined with a stimulant and a little laudanum, by enema.

(3) Sub-cutaneous injection of brandy.

(4) Aromatic spirits of ammonia by the mouth. Champagne is sometimes retained when other things are rejected.

(5) Black coffee and brandy, the stimulant *par excellence*, when it can be retained on the stomach.

(6) Quiet; a horizontal, or more than horizontal position; sleep; assurance that all is over and doing well.

Modern surgery has won *three* great triumphs:

It substitutes sleep for pain.

It averts secondary hemorrhage by the animal ligature.

It prevents fermentation by germicidal applications.

Can we add a *fourth* by stilling the nervous system and averting or diminishing secondary shock?

